# NK/T-cell lymphoma of bilateral adrenal glands in a patient with pyothorax

**DOI:** 10.1186/1746-1596-7-114

**Published:** 2012-08-29

**Authors:** Tomohide Tsukahara, Akira Takasawa, Masaki Murata, Kazuyoshi Okumura, Masato Nakayama, Noriyuki Sato, Tadashi Hasegawa

**Affiliations:** 1 Department of Pathology, Sapporo Medical University, South-1, West-17, Chuo-ku, Sapporo, 060-8556, Japan; 2 Department of Surgery, Tenshi Hospital, North-12, East-3, Higashi-ku, Sapporo, 065-0012, Japan; 3 Division of Clinical Pathology, Sapporo Medical University Hospital, South-1, West-16, Chuo-ku, Sapporo, 060-8543, Japan

**Keywords:** NK/T-cell lymphoma, Adrenal gland, EBV, Pyothorax, Autopsy

## Abstract

Primary lymphoma of adrenal glands is rare, and non-B-cell lymphoma associated with pyothorax is also very rare. Here we report the first autopsy case of non-B-cell lymphoma in bilateral adrenal glands of a 79-year-old woman with pyothorax who had an aggressive clinical course. Immunohistochemically, tumor cells showed CD3+, CD45RO+, CD5-, CD7-, CD4-, CD8-, CD10-, CD20-, CD30-, CD79a-, CD138-, CD56-, granzyme B-, TIA-1+ and ALK-. In addition, tumor cells were strongly EBER1-positive by in situ hybridization. In genomic DNA of tumor cells, T-cell receptor rearrangements were not detected by southern blotting. We finally diagnosed this case as extranodal NK/T-cell lymphoma (nasal type).

**Virtual Slides:**

The virtual slide(s) for this article can be found here: http://www.diagnosticpathology.diagnomx.eu/vs/8050621197741854.

## Background

Lymphoma in the adrenal gland is rare, accounting for less than 1% of non-Hodgkin lymphomas. The features of lymphoma in adrenal glands are: (i) peak age of onset is elderly (mean age of 68 years), (ii) bilateral adrenal glands are involved in 60% of cases, (iii) adrenal failure occurs in 66% of cases, (iv) B-cell type is predominantly observed in 90% of cases and (v) prognosis is poor [1]. On the other hand, pyothorax-associated lymphoma, which is also rare (only 2% of patients with pyothorax), commonly shows the following features: (i) pathogenesis is related to pyothorax resulting form tuberculosis and artificial pneumothorax, (ii) common site of the tumor is around the pleural cavity, (iii) period of onset is more than 20 years, (iv) B-cell type is typical and strongly positive for Epstein-Barr virus, and (v) prognosis is poor [2]. However, non-B-cell type lymphoma occurring in adrenal glands of a patient with pyothorax has not been reported.

Here we report the first case of NK/T-cell lymphoma in bilateral adrenal glands in a patient with pyothorax.

## Case presentation

A 79-year-old Japanese woman presented with cough and bloody sputum in July 2011. She had been followed since 1990 by a pulmonologist under the diagnosis of chronic pyothorax resulting from tuberculosis, and warfarin had been administered under the diagnosis of atrial fibrillation since 2009. There was no obvious evidence of existing immunodeficiency.

**Figure 1:**
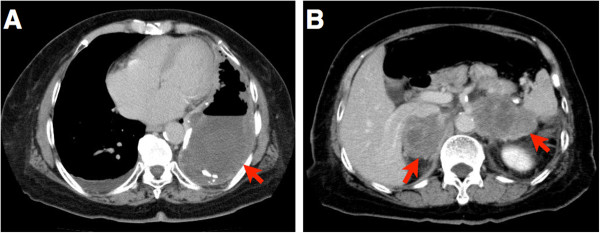
**Enhanced computed tomography scan of the chest and abdomen.** (**A**) Thoracic CT scan displayed large pyothorax and effusion in the left pleural cavity (indicated by a red arrow). (**B**) Abdominal CT scan displayed bilateral adrenal tumors (indicated by red arrows).

At the initial presentation, a large cystic lesion, pleural fluid and bilateral independent adrenal tumors (55 mm x 31 mm on the right side and 57 mm x 32 mm on the left side) were detected by thoracic and abdominal computed tomography (CT) scans, respectively (Figure 1A, 1B). Tuberculosis bacterium was not detected in the sputum using PCR. Serum LDH was elevated to 1,038 U/L. Although use of warfarin was immediately stopped and coagulation therapy was performed using carbazochrome sodium sulfonate hydrate and tranexamic acid, the symptoms were not improved. Nineteen days after initial presentation, bloody sputum was successfully decreased by bronchial artery embolization. The next day, however, hyponatremia (Na: 113 mEq/L) occurred, followed by increases of serum IL-2R (1,185 U/mL) and serum NSE (117.9 ng/mL). An abdominal CT scan showed an increase in sizes of the bilateral adrenal tumor masses (57 mm x 54 mm on the right side and 74 mm x 45 mm on the left side). Two days later, under the diagnosis of adrenal failure caused by the tumors, augmentation of corticosteroid and correction of hyponatremia were started. Serum concentrations of cortisol, sodium and potassium were controlled well. However, seven days after the initiation of treatment, the patient had symptoms of general malaise and chest pain as well as atrial fibrillation with a rapid ventricular response. Serum LDH was increased to 3,650 U/L from 1,038 U/L at the initial presentation. Despite administration of anti-arrhythmic agents, blood pressure decreased to less than 70 mmHg, and then cyanosis and hypouresis occurred. Thirty-three days after initial presentation (11 days after initiation of therapy for adrenal failure), the patient died. Autopsy was performed five hours after death.

## Materials and methods

The autopsy specimen was fixed with 10% buffered formaldehyde and embedded in paraffin. Sections were cut to 2um in thickness and stained with hematoxylin and eosin. Immunohistochemistry was performed using primary antibodies against cytokeratin, vimentin, CD3, CD45RO, CD5, CD7, CD4, CD8, CD10, CD20, CD79a, CD138, CD56, granzyme B, TIA-1, ALK and Ki-67. Ki-67 index was calculated as the ratio of Ki-67-positive cell number to 1,000 tumor cells. In situ hybridization was performed using an anti-sense probe of Epstain-Barr virus (EBV)-encoded RNA 1 (EBER1). Southern blotting for detection of T cell receptor rearrangement in genome DNA of fresh frozen tumor tissue using probes against Jß1 and Jß2 of TCR ß chain and Jγ of TCR γ chain was performed by BML, Inc (Tokyo, Japan).

## Results

### Macroscopic findings

**Figure 2:**
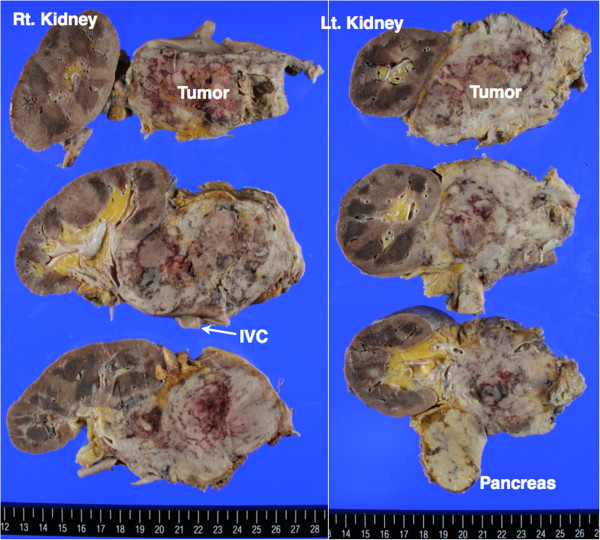
Macroscopic view of bilateral adrenal masses and adjacent organs.

In the left pleural cavity, pyothorax as a large cystic lesion (approximately 16 cm x 7 cm) containing necrotic tissues with pyogenic pleural fluid was detected. No tumoral lesion was detected around the left pleural cavity. In both adrenal glands, tumor masses (90 mm x 65 mm on the right and 85 mm x 60 mm on the left) with necrotic change were detected (Figure 2). The right adrenal tumor had infiltrated into the right kidney and inferior vena cava, and the left adrenal tumor had infiltrated into the left kidney and spleen. Enlarged lymph nodes were not detected anywhere.

### Histologic and genomic findings

**Figure 3:**
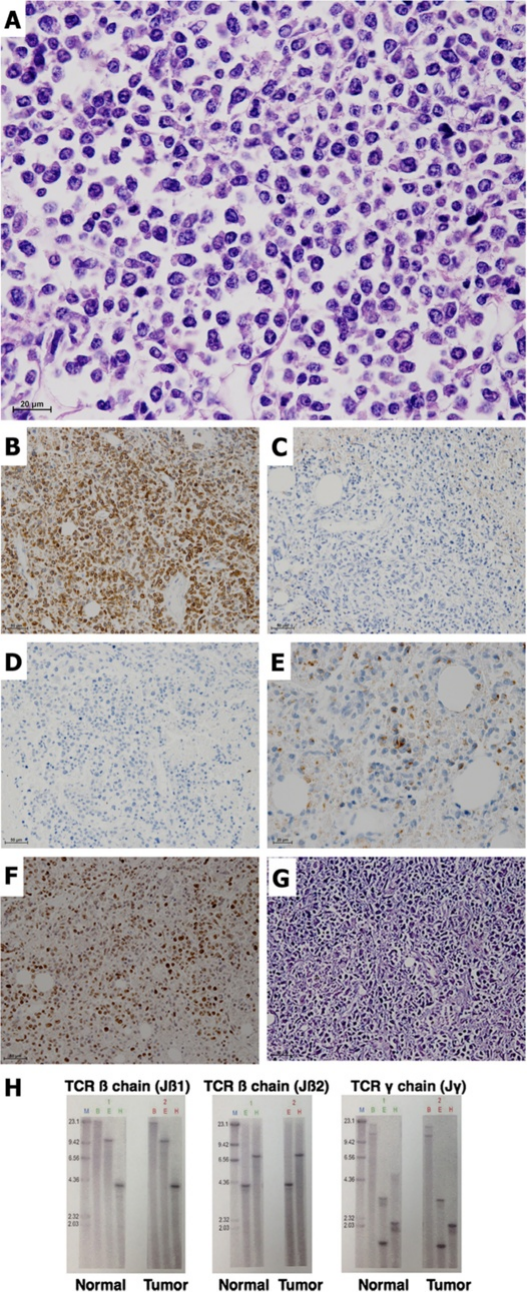
**Microscopic views and T-cell receptor rearrangement of the tumors.** HE (**A**) and immunohistochemistry against CD3 (**B**), CD20 (**C**), CD56 (**D**), TIA-1 (**E**) and Ki-67 (**F**). (**G**) In situ hybridization against EBER-1. (H) Southern blotting to detect T-cell receptor rearrangement in fresh frozen tumor tissue. The first column in each blot indicates the size of the DNA fragment (kbp). Restriction enzymes used were as the follows: E, EcoRI; B, BamHI and H, HindIII.

The cystic wall of the pyothorax consisted of fibrous and hyalinizing tissue with a small population of infiltrating lymphocytes without atypia. No EBER1-positive cells were detected around the left pleural cavity by in situ hybridization. The adrenal tumors contained diffuse middle to large-sized neoplastic lymphoid cells with atypia and mitosis (Figure 3A). Both of the adrenal glands had completely disappeared due to invasion of the tumors. Immunohistochemically, the tumor cells were cytokeratin -, vimentin +, CD3+ (Figure 3B), CD45RO+, CD5-, CD7-, CD4-, CD8-, CD10-, CD20- (Figure 3C), CD30-, CD79a-, CD138-, CD56- (Figure 3D), granzyme B-, TIA-1 cytotoxic granule-associated RNA binding protein (TIA-1) + (Figure 3E) and ALK-. As shown in Figure 3F, Ki-67 was strongly positive in tumor cells (Ki-67 index: 77.0%). Results of in situ hybridization showed that EBER1 was strongly positive in most of the tumor cells (Figure 3G). TCR rearrangements in genomic DNA of fresh frozen tumor cells were not detected in TCR ß and TCR γ chains by southern blotting (Figure 3H).

These findings indicated that the tumor had characteristics of peripheral T-cell origin or natural killer (NK)/T-cell origin. The findings of CD3+, CD45RO + and CD56- could indicate peripheral T-cell lymphoma. In contrast, the findings of TIA-1+, EBER1+ and no TCR rearrangement could indicate NK/T-cell lymphoma. Considering the findings of TIA-1+, EBER1+ and no TCR rearrangement, histological type of the tumor was finally diagnosed as CD56-negative extranodal NK/T-cell lymphoma (nasal type).

## Discussion

This is the first case of non-B-cell lymphoma in bilateral adrenal glands in a patient with pyothorax. Tumor cells had the characteristics of both peripheral T-cell lymphoma and NK/T-cell lymphoma. In addition, no tumor lesions were detected around the pyothorax. The tumor showed highly malignant characteristics and showed an aggressive clinical course.

Commonly, adrenal lymphoma shows histological characteristics of B-cell type, mainly diffuse large B-cell lymphoma (DLBCL). Therefore, this case of non-B-cell lymphoma is a rare case. Six cases of peripheral T-cell lymphoma and five cases of NK/T-cell lymphoma have been reported [3,4]. On the other hand, most pyothorax-associated lymphomas have shown findings of DLBCL occurring in the pleural cavity in addition to strong positive findings of EBV. Nine cases of peripheral T-cell lymphoma associated with pyothorax have been reported [5-13], but no cases of NK/T-cell lymphoma associated with pyothorax have been reported. Only one case of lymphoma occurring in both adrenal glands in a patient with pyothorax has been reported [14]. In that case, a small lesion of lymphoma was also detected around the pyothorax lesion, and the histological type was diagnosed as DLBCL. Thus, non-B-cell lymphoma in adrenal glands in a patient with pyothorax such as the present case is an extremely rare condition.

For differential diagnosis, (i) “peripheral T-cell lymphoma, not otherwise specified”, (ii) “extranodal NK/T-cell lymphoma (nasal type)” and (iii) chronic active Epstein-Barr virus infection (CAEBV) were considered. As described above, the tumor had characteristics of both peripheral T-cell and NK/T-cell lymphomas. The findings of CD3 + CD45RO + CD56- could indicate peripheral T-cell lymphoma. On the other hand, the findings of cytotoxicity-associated molecule TIA-1+, no TCR rearrangement and EBV + could indicate NK/T-cell lymphoma. The present case could not strictly be categorized into either. However, considering the findings of TIA-1+, EBER1+ and no TCR rearrangement, we finally diagnosed this lymphoma as extranodal NK/T-cell lymphoma (nasal type). Recently, Miles et al. reported a case of CD56-negative extranodal NK/T-cell lymphoma [15]. In that case, neoplastic lymphoid cells expressed CD3, TIA-1 and EBER1 with an unusual lack of CD56. In addition, the patient had no typical symptoms of infectious mononucleosis-like illness, hypersensitivity to mosquito bites or other symptoms supporting CAEBV [16].

Our patient died only 33 days after initial presentation. Generally, the prognosis of both peripheral T-cell lymphoma and NK/T-cell lymphoma is poor. Nonnasal NK/T-cell lymphoma, such as this case, shows an aggressive clinical course. Chen et al. reported that the mean survival period of nonnasal NK/T-cell lymphoma patients was 3.5 months (from 1 week to 3 years) after initial presentation [17].

In pyothorax-associated lymphoma, transformation of lymphocytes caused by EBV infection and proliferative stimulation via inflammatory cytokines including interleukin-6 in the microenvironment of chronic pyothorax might be the major cause of tumorigenesis [8]. In a narrow sense, the association between pyothorax and the present lymphoma of adrenal glands is still unknown because no tumor lesions were detected in the pleural cavity. Obviously, there is a possibility that the pyothorax existed incidentally. Nevertheless, the possibility that both the long-standing pyothorax lesion and EBV infection contributed to the tumor-initiating ability of tumor cells in the present case cannot be ruled out. Asakage et al. reported a case of EBV-positive T-cell lymphoma of the stomach in a patient with pyothorax. In that patient, no tumors were detected around the pleural cavity, as in the present case [5].

## Conclusion

We have reported the first case of CD56-negative extranodal NK/T-cell lymphoma in bilateral adrenal glands in a patient with pyothorax. The tumor displayed highly malignant characteristics with a distressful clinical course. There is a possibility that chronic inflammation in the microenvironment of the pyothorax and EBV infection contributed to the tumor-initiating ability of tumor cells in the present case.

### Consent

Written informed consent was obtained from the family of the patient for publication of this case report and any accompanying images. A copy of the written consent is available for review by the Editor-in-Chief of this journal.

## Competing interests

The authors declare that they have no competing interests.

## Authors' contributions

TT, AT and MM performed the autopsy and assessed macroscopic and microscopic findings. KO and MN treated the patient and contributed to acquisition of clinical data. TT and AT drafted the manuscript. NS and TH participated in the coordination. All authors read and approved the final manuscript.
